# DIVISION OF EXTRAMURAL ACTIVITIES

**DOI:** 10.1093/geroni/igaa057.3011

**Published:** 2020-12-16

**Authors:** Kenneth Santora

**Affiliations:** National Institute on Aging, Bethesda, Maryland, United States

## Abstract

Dr. Santora will discuss research priorities for the Division of Extramural Activities. He and his team will in addition be available for small group discussions

